# Strictures on the Review of an Essay on Medical Economy, in the Med. and Phys. Journal for April 1814

**Published:** 1814-06

**Authors:** 


*For the Medical and Physical Journal.*

### *Strictures on the Review of an Essay on Medical Economy, in the Med. and Phys. Journal for April* 1814.

DURING a period of years which I passed remote from the busy scenes which now surround me, the Medical and Physical Journal brought me almost all the intelligence which I received of the progress of the medical art in Europe, and of occurrences connected with it. As a companion, therefore, with which I have conversed much and become familiar in my solitude, I continue to feel an interest in it; and as I would frankly warn any other friend of a mistake into which he might accidentally fall, so I take the liberty of sending you a few observations on an article which has been admitted into the last Number of the Journal. It is the review of a work which has for title, "An Essay on Medical Economy; comprising a sketch of the state of the Profession in England, and the outlines of a plan calculated to give to the Medical Body in general an increase of usefulness and respectability."

This work I procured in consequence of seeing it noticed in your Journal, but I have read it with a degree of pleasure which the tenour of that notice did not lead me at all to expect from it. I was surprised, indeed, to see the reviewer pay almost his highest compliments to the talent and philosophy of the author, and still find, as it appeared, so much reason to object to the performance. On examining the book, I discovered, however, the reason of this apparent inconsistency. The reviewer has in part misconceived the author's opinion and object, either from careless perusal or pre-occupation in some other pursuit. Falling into error himself, he naturally carries others along with him; and had I been, as most of your readers are, busily employed in practice, instead of seeking for a time to repair injured health by varied occupation and amusement, it is probable that the mis-statement would have prevented me altogether from looking into the book, and consequently would have deprived me of the information and pleasure which I have had in reading an essay upon a very important subject, and which I account almost perfect in its kind.

The sum of the author's reasoning and suggestion is, that the medical art is much less useful to the community at present, than a little legislative regulation might make it. He states the fact, that of the great body of practitioners a very small number comparatively have received complete education; and the law carelessly allowing unqualified men to assume the garb and title of the profession, in a way which renders it impossible for the public to distinguish between them and their well-informed brethren, unprincipled effrontery often obtains the reward of modest worth and excellence. Hence medical education, not bringing to its possessors advantages by any means proportioned to its great expense and difficulty, common minds do not desire it, both from motive of interest, and from the natural wish to save trouble wherever it can be done. The law may thus be said to establish a low rate of qualification in the profession; and to this cause the author attributes the slow progress which medical science has made compared with others. To remedy the defect, he proposes that only physicians, or men of complete education, be allowed to practise. He enters into minute and clear detail to shew the easy practicability of the change with advantage to all parties; the body being divided into orders of seniority, with difference of fee, to suit the ability to pay of all classes of society, while its general respectability would be much increased, by well-educated gentlemen being substituted for the heterogeneous mass of apothecaries of the present day. Were the plan to be put in force immediately, while the man who could not prove his title to confidence would be totally excluded, the well-educated apothecary, and there are many such, would change his appellation and continue to practise.

Such is the idea of the work which remains to me after a second and attentive perusal. The reviewer makes two objections, however, but curiously enough, not to any thing which the book contains, but to what he erroneously supposes it to contain, and founded on these he forms his judgment. 1st, He accuses the author of stigmatising the profession as wanting ability, when in fact he only laments that the ability in it is not called into exertion for the improvement of the art, owing to the unfavourable circumstances above alluded to. 2nd, He states, "*the leading feature of the plan is, to convert apothecaries into physicians:*" the truth being that it is proposed to substitute physicians for the present apothecaries, and to abolish the name of the latter, with the abuses which it has covered. He thinks also, that the junior physician would be degraded by the smallness of his fee. The author says on this subject, that the junior may be considered as bearing a similar relation to the senior, to that which the midshipman bears to his admiral. The midshipmen are young gentlemen, but submit to all the drudgery assigned them without complaint: they feel that they are on the same road with their admiral, qualified to bear him company in the hour of relaxation, and fairly entitled to look forward to the enjoyment of all his dignities as the full reward reward of their services. I was perhaps not so much struck, however, by these marks of little care in the examination, as by the reviewer's assertion that no reform is wanted at all. This may be true as applied to the situation and prospect of some individuals of the profession; and indeed I am sure the writer must here have been for a moment under the impression, that he was advocating his own, or some friend's private interest, for he could never have believed it, as the public is concerned. We find in every part of the metropolis and kingdom men undertaking the management of disease in all its kinds and stages, whose education went hardly beyond the art of making a pill or compounding a mixture, and who of course, in undertaking the practice of medicine, act as that colour-grinder would do, who seizing the pencil of Raphael or Reynolds, should offer himself to furnish decoration for a royal gallery. We find this, and we know that not one in ten of those who practise have received a complete education, or can merit confidence where science is wanted. Can we say with propriety, then, "the public, and with great reason, seems contented with the skill, talent, integrity, and general conduct of the various practitioners whom they patronise?" And again, "the three grades of physicians, surgeons, and apothecaries, are adequate to every good purpose?"

Government, as the enlightened and affectionate father of a numerous family, is careful to guard its children against many dangers and impostures to which their inexperience or unavoidable want of information exposes them; it examines and authorises, for instance, all pilots, engineers, naval officers, &c. &c. why should not the same care be extended to all possible cases, to one particularly where all the members of the community are concerned, and in which they are so much exposed to deceit—to the practice of medicine? If to prevent pain to relatives and to the lovers of justice, twenty malefactors are allowed to go unpunished rather than one innocent man shall suffer, must nothing be done to spare to survivors that needless agony which we so frequently witness on account of premature death from ignorant mistake in medical treatment? But two days ago I suffered more on this account than I had believed it possible for me to feel for a person so lately known to me. The wife of an old acquaintance of mine has in this way been suddenly and unexpectedly left a widow with several children. A vile apothecary was nursing with poultices what he called an abscess in her husband's leg; when in a moment of exertion it burst, and the unhappy man's life fled with his blood from a ruptured aneurism.

COLLEC-

